# Impact of a simulation-based education approach for health sciences: demo, debrief, and do

**DOI:** 10.1186/s12909-023-04655-w

**Published:** 2023-10-10

**Authors:** Elizabeth Orsega-Smith, Tara Leonard, Laurie Ruggiero, Nicolette Amato, Jamie O’Hara

**Affiliations:** Department of Health Behavior and Nutrition Sciences, University of Delaware, 26 North College Avenue, De 19716 Newark, USA

**Keywords:** Simulation based exercise, Peer learning, Skills-based practice, Health coaching

## Abstract

**Background:**

Skill-based practice (e.g., communication skills) is important for individuals to incorporate into students' learning and can be challenging in large classes. Simulation-based education (SBE) is a method where students can learn and practice skills in a safe environment to use in real world settings with assistance of peer coaching. The COVID-19 pandemic presented challenges to providing students with sufficient SBE. The purpose of this paper is to: a.) describe a SBE approach for health coaching referred to as “Demo, Debrief, and Do” (DDD), b.) discuss how this approach became important in COVID-19 classroom experiences, c.) describe the impact of DDD activity on students in a health sciences curriculum. DDD is a collaborative activity where graduate health coaching students *demonstrate* coaching skills, *debrief* their demonstration, and support undergraduate students to demonstrate (or *do*) their own coaching skills in a small virtual online setting.

**Methods:**

Qualitative feedback from 121 undergraduate students enrolled in 3 sections of a behavior change strategies course and quantitative surveys to examine their confidence in applying the skills and overall satisfaction with DDD were gathered.

**Results:**

The overall average confidence level following the lab was 31.7 (0–35). The average satisfaction level following the lab was 23.3 (0–25 range). The most common highlight of this DDD experience described was observing the coaching demonstration (i.e., demo), followed by the feedback (i.e., debrief), and the practice (i.e., do).

**Conclusion:**

The (DDD) simulation approach fulfilled an educational need during the COVID 19 pandemic and filled a gap in offering SBE opportunities for both graduate and undergraduate students while learning effective client-communication skills health coaching delivery.

**Supplementary Information:**

The online version contains supplementary material available at 10.1186/s12909-023-04655-w.

## Background

Skill-based practice is important for individuals to utilize in health-related fields and may be critical to incorporate into students' learning. However, educating students to learn skill-based techniques can be challenging, especially in large classes given the complexities of implementing hands-on practice with large groups. Most of the literature focused on large class sizes with skill-based techniques is in medical related fields and generally incorporates “flipped classroom” techniques followed by the use of simulation-based experiences [1, 2]. Simulation-based education (SBE) is another method by which students can learn and practice skills that they would use in real world settings. SBE is a practice in which students undergo guided experiences where they take on various roles (client, health care professional, family member) in acting out a case study experience [3]. It may be used in situations where participants may have hands-on experience in an environment where learners may feel more comfortable practicing with reduced fear of making mistakes [4].

SBE activities have shown promise in increasing competencies in healthcare delivery in both undergraduate and postgraduate students [3]. In a recent review article [5], researchers determined that SBE included the following as best practices: simulation design and delivery (i.e., interactivity and repeated practice), resources (i.e., facilitator competency), and curriculum related integration and planning (e.g., curriculum application, opportunities for practice). The authors further suggest that SBE should focus on these best practices [5].

The literature suggests that SBE using peer coaching may be beneficial for student learning. Ickes and Mcmullin [6] reported successful teaching outcomes from utilizing graduate students in the health coaching field. In their example, 15 students were enrolled in a graduate health promotion and behavior change course that focused on health coaching techniques. The health coaching students were paired up with participants in a campus-based physical activity course designed specifically for obese college students. As a result of fostering a coaching relationship with these undergraduates, the graduate students reported improvements in self-efficacy for health coaching skills, knowledge scores, and comfortability with the skills necessary to be a coach [6]. Although literature has discussed the challenges for students in health sciences to transfer the classroom knowledge into practice [7], peer coaching is one method to enhance peer learning and increase skill learning and professional development as a form of SBE. For the purpose of this paper, peer coaching is described as peers participating in a demonstration, observation, discussion and feedback experience to learn with and from one another. The use of peer coaches is well-aligned with social cognitive theory of learning and can help students to increase their self-efficacy [8] in implementing these skills [7].

### Teaching and learning challenges posed by COVID-19 Pandemic

The COVID-19 pandemic presented new challenges to providing students with sufficient simulation-based education. The pandemic forced a quick pivot to online learning. This was a difficult transition for both educators and students who had to adapt to teaching and learning in virtual environments [9, 10]. Regarding university level students, this transition posed a greater learning challenge because of the diverse populations, various student learning needs, and limited access to technology to facilitate successful online learning [9]. Furthermore, some of the students who felt most productive with face-to-face instruction and small group assignments may have felt a void in their overall learning experience. In several cases, students expressed needing real-time interactions with their professors and peers to assist with learning techniques [11]. A study by Adnan & Anwar [11] found that 42.9% of college students felt they struggled to complete group projects in distant learning formats. The need for real-time interactions during the transition to virtual learning environments posed the question of how to incorporate simulation-based education into curriculums.

### Potential solutions

Researchers have examined potential solutions to educate students using SBE throughout the pandemic [12–14]. In Canada, a group of educators worked with master’s in social work students to implement “Virtual Practice Fridays” [13]. This experiential learning technique consisted of splitting a large class into groups of 10 students by utilizing virtual breakout rooms. During the sessions, the students practiced the role of a social worker working with a client. After completing their role as the social worker, the students received feedback from both their faculty and peers [13]. In this case, the students processed case notes, reviewed recordings, and completed reflections on their client interactions. Similarly, in a medical school setting, Jeong and colleagues [12] implemented virtual peer teaching into their curriculum to work on skills related to patient education. In this example, teaching guides outlining what should be covered were created and implemented throughout each session. These guides were a way to maintain fidelity of the sessions. Jeong and colleagues [12] planned to continue administering these experiential techniques in their curriculum after returning to in-person learning, as they believed this style of teaching can benefit faculty, students, and peer teachers. Malone [14] also used a virtual platform to facilitate learning during the pandemic in their nurse residency program. The use of a virtual platform in this setting allowed for nursing students and faculty to interact, while the small group settings used for case study reviews provided enhanced opportunities for feedback and interactions from peers. Survey results emphasized the importance of virtual peer interactions as 95% felt they were helpful. In multiple health science disciplines, utilizing small groups in a virtual setting allowed students to continue to progress in their knowledge while providing opportunities to connect with their peers and mentors.

The purpose of this paper is to: a.) describe a simulation-based education approach for health coaching referred to as “Demo, Debrief, and Do”, b.) discuss how use of this approach became more important in COVID-19 classroom experiences, and c.) describe the impact of this Demo, Debrief, and Do activity on both the graduate and undergraduate students in a health sciences curriculum.

### Health behavior science course context

The overall context for this study was an undergraduate Health Behavior Science course focused on teaching junior or senior undergraduate level students the application of behavior change strategies often completed through case studies and practice scenarios. The graduate component incorporated graduate health coaching students within this institution’s Health Coaching Certificate program. In Health Coaching training, emphasis is placed on individual and group-oriented coaching scenarios through personal practice and observation using simulation-based education approaches. As part of the training process, students practice creating a safe space for client interactions as well as debriefing a client-centered interaction with peers as well as clients [15, 16].

The standards for training health coaches in the certificate program are set forth by the National Board for Health and Wellness Coaches (NBHWC). Health and Wellness Coaches work one on one or with groups of individuals pursuing improved overall health. This is achieved through the individual's partnership with a health coach who may assist in the creation of self-directed health behavior changes to support sustainable and long-term health and wellness [17].

The health coaching skills included motivational interviewing (a collaborative style of communication between a practitioner and client, appreciative inquiry) a strength-based approach to creating lasting positive change, goal setting, evoking, guiding, and supporting clients’ decisions around their health behaviors to reduce the impact of chronic disease and improve their health and well-being [14, 18, 19].

### Instructor challenge

The instructors of both the graduate and undergraduate courses were also challenged in teaching these health coaching skills in a larger class size. Due to the large class size, it was difficult for the instructor to gauge student’s ability to practice the communication styles being taught while providing specific feedback in a timely fashion.

### Rationale for graduate students working with undergraduate students

Lecture-based teaching alone does not offer the experiences and skills needed to prepare students for a career in health and wellness coaching [20]. Informal student feedback obtained after course completion indicated that the graduate students desired more opportunities for SBE learning experiences. To address the needs of the students, combining graduate and undergraduate students in this format in which both student populations could obtain these hands-on learning opportunities helped address the desires of the students as well as the challenges of instruction during COVID. This format allowed demonstration of high-quality coaching, time to debrief the demonstration with the instructor and peers, and time to practice coaching in front of the instructor and peers. Additionally, the format also supported opportunity to practice proficiency in the necessary coaching skills to work with actual clients, as it is suggested to practice immediately after observing, practice often, and practice in different modalities to adequately refine skill sets, all in a controlled environment [3, 21, 22].

### Rationale for undergraduate students

Initially, it was difficult for the undergraduate students to gain a clear understanding of the micro-skills necessary for developing health coaching competencies. Ickes & McMullin [5] note the complexities of teaching health coaching skill sets while ensuring regular practice and feedback from experienced health coaches. The authors highlight specifically the skills of, “fostering autonomy, expressing empathy, intrinsically motivating individuals, and suggesting strategies to improve self-efficacy” and acknowledge that they are developed over time and with generous opportunity to practice [6]. Previously in this course, videos, class activities, and practice with peers were the most common strategies used by instructors to teach undergraduates coaching skills. Unfortunately, the video demonstrations were long, which made it difficult for students to focus on the sections needed to learn the skills. The large number of students enrolled in the course made it difficult for them to experience personalized guided practice, feedback, clarification, and discussion about the variety of health coaching scenarios they were practicing. For some individuals, group work can be intimidating to practice skills while experienced faculty and peers are present. To increase learning proficiency and address these concerns, the instructors brainstormed ideas and decided to have health coaching graduate students demonstrate the skills for undergraduates, as well as facilitate class discussions and SBE in smaller groups. It was felt this approach would support a more relaxed collaborative environment, encourage greater engagement, and facilitate comfort in asking questions.

### “Demo, Debrief and Do”: Description, development, and implementation

The “Demo, Debrief and Do” is a collaborative activity in which graduate health coaching students *demonstrate* coaching skills, *debrief* their demonstration with the undergraduate students within the group, and support the undergraduate students as they work in pairs to demonstrate (or *do*) their own version of the coaching skills in front of the small group. Through the graduate coaching demonstration, the undergraduate students observe a live interaction between a coach and client. The graduate student coaches may pause the demonstration to debrief in the moment the skills utilized during the coaching conversation. They additionally debrief after the conclusion of the demonstration which allows continuous, rich discussion about the interaction as well as open conversation about strategies (tips and tricks) to support undergraduate learning and engagement. Finally, when the undergraduate students take turns as the coach and client, immediate feedback and opportunities to “re-coach” are offered in the moment which supports improved individual and group learning. See Table 1 for a thorough description of Demo, Debrief, and Do.

**Table 1:** Demo, debrief, and do description

Demo	Graduate Students	• Graduate student demonstration of the undergraduate case study showcasing coaching micro skills (building rapport, use of MI skills and goal setting) • Sessions may have “starts and pauses” to allow graduate students the opportunity to explain a concept after demonstrating it in real time
Debrief	Graduate Students Undergraduate Students	• Graduate students lead discussion and offer strategies (e.g., tips and tricks) for improving micro skills by pausing to debrief both during the demonstration and again at the conclusion of the demonstration • Undergraduate students prepare to demonstrate micro skills with peers by asking questions and taking notes
Do	Undergraduate Students	• Undergraduate students in peer pairs (i.e., client and coach roles) practice micro skills in front of graduate students and peers • Sessions may have “starts and pauses” to allow students the opportunity to “re-coach” and gather real-time feedback and suggestions. Either graduate or undergraduate may pause session for clarification or suggestions

The development of the Demo, Debrief, and Do approach was an iterative process. Initially, in the Fall of 2019, the technique started with two graduate coaching students visiting the undergraduate classes and demonstrating a live coaching interaction on a few occasions during the semester. At that point, there was not a specific case study assignment. This was primarily a demonstration of a general role-played coaching interaction with a bit of engagement from students practicing the skills demonstrated and participating in a question/answer session. Therefore, this iteration of Demo, Debrief, and Do was not structured and did not include demonstrating a specific set of coaching skills from beginning to end. One of the themes from the feedback obtained from both the undergraduate and graduate students during this experience was that they wanted more similar simulation-based education opportunities. As COVID-19 restrictions related to in person learning continued to be in place for the Fall of 2020, offering an opportunity for both groups began to surface as a viable solution to provide more simulation-based education class sessions while also filling the need for graduate students to get sufficient training hours in health coaching. In the fall of 2020, the first small group sessions with graduate student coaches occurred virtually in the undergraduate course. During the class session, graduate students demonstrated a behavior change case study for the full class designed to showcase specific coaching skills and then discussed the demonstration in small groups (using break out rooms) with the undergraduate students in a safe environment to help reduce the fear of completing the activity in front of a large group. This specific case study was used as a course skill demonstration assignment in which the undergraduate students created their own video demonstrating the skills that were taught and practiced during the session.

### Research questions

The research component of this paper focuses on addressing the following questions:

1. What is the impact of the Demo, Debrief, and Do simulation-based education approach on undergraduate students’ learning, satisfaction, and confidence in the microskills taught?
2. What is the impact of leading the SBE experiential activity on health coaching graduate students?

## Methods

### Design

The study used a convergent mixed methods design. A parallel cross-sectional survey approach was used to gather qualitative feedback from undergraduate students on the lab experience and quantitative responses to examine their confidence in applying the skills taught, and overall satisfaction with the experience. In addition, qualitative feedback was informally gathered from graduate health coaching students and instructors.

### Participants

The study focused on undergraduate students enrolled in a course that teaches students about how to apply behavior change strategies (e.g., Motivational Interviewing techniques and micro skills). Motivational interviewing (MI) is a central person-centered behavior change framework covered in this course. Motivational interviewing “is a collaborative, goal-oriented style of communication with particular attention to the language of change. It is designed to strengthen personal motivation for and commitment to a specific goal by eliciting and exploring the person’s own reasons for change within an atmosphere of acceptance and compassion. ([23], p. 29). “OARS” is an acronym for a set of micro-skills that are central to MI and represent open questions, affirmations, reflections, and summarization. In brief, open questions are those that “draw out and explore the person’s experiences, perspectives, and ideas” (e.g., How would you like things to be different?) and avoid closed questions that obtain limited (e.g., How often do you _____?) or one-word (e.g., yes/no) responses; affirmations are used to acknowledge a person’s “strengths, efforts, and past successes” (e.g., You were really resourceful in your efforts to quit smoking in the past) and “help to build the person’s hope and confidence in their ability to change” (e.g., You have shown determination and skillful problem solving so far; these will help you reach your new healthy lifestyle goals); reflections “are based on careful listening and trying to understand what the person is saying, by repeating, rephrasing or offering a deeper guess about what the person is trying to communicate” (e.g., It sounds like you…); and summarization may be used throughout and at the end of an interaction (e.g., You have shared a lot of important information, let me summarize and see if I understand so far…) and “ensures shared understanding and reinforces key points made by the client” [24].

Students are taught to use these micro-skills, along with other concepts and strategies from MI and other health behavior change theories in this behavioral science course. OARS micro-skills were a main component included in this DDD simulation. A detailed description of Motivational Interviewing is beyond the scope of this paper. A variety of resources, textbooks, and research papers are available describing this approach and the evidence supporting it [25, 26].

This behavior change course is a required course for all students in the Health Behavior Science Bachelor of Science degree program at a mid-Atlantic area University. Participants were junior or senior year students enrolled in three sections of this behavior change strategies course delivered from Fall 2020 to Spring 2021. In total, 121 undergraduate students were enrolled in the three sections and there were 78 responses to surveys and 113 responses to open-ended questions.

All procedures were reviewed and approved by the institution’s Institutional Review Board; and the research was exempt from federal policy requirements for the protection of human subjects.

### Evaluation methods

#### Undergraduate

##### Satisfaction and self-confidence quantitative measure

The “Student Satisfaction and Self-Confidence in Learning Scale” was used to examine satisfaction and self-confidence regarding the DDD lab experience [27, 28]. This is a 13-item questionnaire with two dimensions, i.e., satisfaction with current Learning (5-items; range = 0–25) and self-confidence in learning (8-items; range = 0–40). Five items were slightly modified to reflect the students’ satisfaction specific to the demo, debrief and do labs, such as replacing “medical curriculum” with “health coaching skill set”. Items pertaining to satisfaction with learning included “The methods …were helpful and effective.” I enjoyed how the instructor taught the simulation.” Items referring to self-confidence in learning included “I know how to use simulation activities to learn critical aspects of these skills.”, “I am confident that I am mastering the content of the simulation activities…”. A 5-item Likert response scale is used with responses from “strongly disagree” (scored 0) to “strongly agree” (scored 5). An average score and standard deviation were calculated for of the two dimensions where higher scores indicate higher satisfaction and higher self confidence in learning. This measure was used with the two sections offered in the Fall 2020 semester.

##### Qualitative survey questions

The qualitative survey questions were developed by the researchers to address the question: “What is the impact of the Demo, Debrief, and Do simulation-based education approach on undergraduate students’ learning?” A qualitative survey (Supplemental File 1) was completed after the DDD simulation activity that asked the following questions: *(a) What was the highlight of working with the graduate students for you? and (b) How did your coaching improve by working with the graduate students?*

##### Qualitative data coding and summarization

The qualitative data from the two survey questions was reviewed multiple times by the coding team, including 2 graduate students and two faculty, to identify codes. A conventional inductive content analysis approach guided this process [29]. The team met over the course of several weeks to consolidate and streamline the coding process. First, each team member independently reviewed the student responses and highlighted themes. After each member finalized themes independently, the team met and compared their themes. Team members agreed upon similar themes and used these to finalize codes and create a codebook. The code book was used to guide summative content analysis of student responses and report findings. In total, five team members used the codebook to code the responses from each of the two qualitative survey questions for the first two simultaneously delivered sections of the course. For the third subsequent course section, only two coders reviewed the qualitative questions to code the data since the codebook was previously developed. A third coder participated, when needed, to resolve inconsistencies of the first two coders for this course section. All coders were paper authors. Themes are listed and described in Table 2.

**Table 2:** Undergraduate student qualitative feedback about the demo, debrief, and do simulation

What was the highlight of working with the student Practicum Health Coaches for you?			
Themes	Theme Descriptions	Frequency	Sample Quote
Demo *(i.e., observing the graduate student coaching interaction)*	The student discussed the observation of the graduate students live or recorded demonstration of the health coaching interaction	52	“Just observing their approach helped me learn and see different ways of health coaching that I can incorporate myself.”
Debrief *(i.e., receiving feedback on their own coaching interaction; tailored/individualized feedback from the graduate students)*	The student discussed live feedback during practice and debriefing without mentioning the key words *information, tips, advice, or guidance*	39	“Highlight to working with the practicum students was getting really personalized feedback when practicing doing the coach.”
Do *(i.e., practicing their own coaching interaction)*	The student discussed the experience of practicing being the coach or the client during the DO portion of the DEMO-DEBRIEF-DO	38	“Being able to practice coaching in front of them, and then they were able to guide us on the right path.”
Awareness of Health Coaching & the Structure of a health coaching interaction	The student discussed gaining awareness of the structure, format, flow, method of the health coaching interaction	13	“Watching this really helped me get an idea of what to expect and how a natural flow of conversation may occur with a client.”
General Information/Tips/Advice/Guidance	The student indicated one of the key terms, *information, tips, advice, and/or guidance,* being provided to them by the graduate students	18	“Getting their advice on what to do when working with a client.”
Increased Confidence	The student discussed improvements in their confidence	6	“They are also students in the master’s program, so they understood what it was like to be new at this and were great at giving us ideas and helping us build our confidence when we practiced the coaching sessions as well.”
Small Group Interaction	The student discussed smaller group interactions in some way being a highlight in comparison to the large class	1	“With the smaller group, I definitely felt better about talking and having someone on one help with the health coaching, so I really loved being in that small group environment with that extra help.”

### Health coaching graduate students

The qualitative data assessing the graduate student experience was obtained through email. Two reflection questions were sent to each graduate student, and they submitted their responses through email. The questions included: 1) What was the highlight of working with the undergraduates for you?; 2) How did your coaching improve by working with the undergraduates? Completion of the graduate student survey was optional. A total of 4 of 8 graduate students completed the follow up reflection questions. Once the students submitted their responses, a content analysis was conducted by 2 team members; themes were compared, inconsistencies were discussed, and final themes/notable quotes were determined. The team members chose a unique quote from each graduate respondent to represent the breadth of themes. Furthermore, the team members omitted responses from the graduate students that were repetitive (See Table 3).

**Table 3:** Graduate student qualitative feedback about the demo, debrief, and do simulation

Qualitative Questions:	Selected Graduate Student Responses:
#1: What was the highlight of working with the undergraduate students?	• “I would see specific students take our feedback and improve on their delivery in the next DDD. I was impressed by the questions they asked and how most of the students were engaged in the process.” • “Getting a chance to gain real experience and receive constructive feedback from both clients and professor.”
#2: How did your coaching improve by working with the undergraduate students?	• “The undergraduates provided me the opportunity to reflect on my style of coaching, and to acknowledge the different coaching styles that different people have, and to find ways to accommodate that in different settings/with different clients.” • “It helped me feel more secure/confident with the whole coaching process. Working with them also helped give room to figure out my coaching style.”

## Results

### Undergraduate results

#### Student satisfaction and self-confidence in learning scale

The overall average confidence level following the lab was 31.7 (SD = 2.8; possible scale range = 0–35). The average satisfaction level following the lab was 23.27 (SD = 2.3; possible scale range = 0–25). Overall, these composite scale levels are high indicating that students felt satisfied with the experience and confident in their learning (see Table 4 for detailed summary of Student Satisfaction and Self-Confidence in Learning Scale).

**Table 4:** Summary of student satisfaction and self-confidence in learning scale

Subscale or Question	Mean (SD) [range]
The teaching methods used in this simulation were helpful and effective. (Satisfaction Question)	Mean: 4.75 ± .45 Range: 3–5
The simulation provided me with a variety of learning materials and activities to promote my learning in the coaching skill sets (Satisfaction Question)	Mean: 4.59 ± .59 Range: 2–5
I enjoyed how my instructor taught the simulation. (Satisfaction Question)	Mean: 4.67 ± .60 Range: 2–5
The teaching materials used in this simulation were motivating and helped me to learn. (Satisfaction Question)	Mean: 4.67 ± .57 Range: 2–5
The way my instructor(s) taught the simulation was suitable to the way I learn. (Satisfaction Question)	Mean: 4.54 ± .7 Range: 2–5
Satisfaction with Current Learning Subscale	Composite Mean: 23.27 SD: 2.3 Range: 10
I am confident that I am mastering the content of the simulation activity that my instructors presented to me. (Confidence Question)	Mean: 4.12 ± .69 Range: 2–5
I am confident that this simulation covered critical content necessary for the mastery of my coaching skill set)	Mean: 4.58 ± .56 Range: 2–5
I am confident that I am developing the skills and obtaining the required knowledge from this simulation to perform necessary tasks (in working with clients)	Mean: 4.48 ± .61 Range: 2–5
My instructors used helpful resources to teach the video (demo and debrief)	Mean: 4.71 ± .56 Range: 3–5
It is my responsibility as the student to learn what I need to know from this simulation activity	Mean: 4.57 ± .56 Range: 3–5
I know how to get help when I do not understand the concepts covered in the simulation	Mean: 4.66 ± .54 Range: 3–5
I know how to use simulation activities to learn critical aspects of (coaching skills)	Mean: 4.49 ± .60 Range: 3–5
Self-confidence in Learning Subscale	Composite Mean: 31.7 SD: 2.8 Range: 11

#### Qualitative feedback

**Fig. 1:**
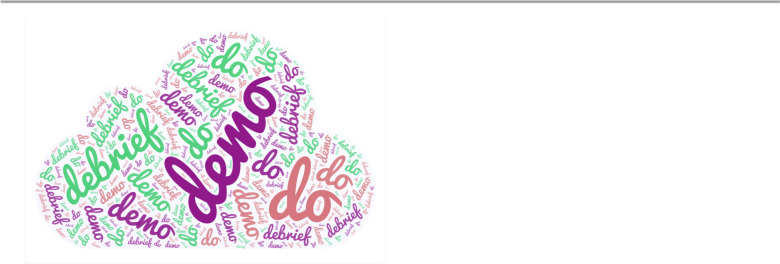
Word cloud of three major themes: demo, debrief, and do

The themes titles, descriptions, frequencies and sample quotes are shown in Table 2. As can be seen in this table, regarding the highlight of the student experience, the most common highlight described was observing the coaching demonstration. A representative quote is “Just observing their approach helped me learn and see different ways of health coaching that I can incorporate myself.” The second and third most common themes were “debrief” and “do” and were reported in similar frequency (See Fig. 1). The debrief comments focused on the feedback they received from coaches on their own role-played coaching interaction. A representative quote is “Highlight to working with the practicum students was getting really personalized feedback when practicing doing the coaching.” The “do” feedback focused on students’ experiences with completing their own role-played interaction. A representative quote is “Being able to practice coaching in front of them, and then they were able to guide us on the right path.” Other common themes included their awareness of the health coaching approach more generally and getting general information (e.g., tips, advice, guidance). In response to the question about the areas they felt improved based on the DDD experience, the most common skill-based themes were learning to be “client-centered” and feeling more confident in their skills. Also, building rapport and maintaining a comfortable flow during the interaction were also common themes. Regarding the themes associated with the mechanisms that impacted their improvement, the most frequently reported theme was receiving feedback or guidance from the coaches. The other common themes were practice, demonstration by the coaches, and the debrief (e.g., getting advice, tips, suggestions on how to reframe questions using open-ended questions, creating appropriate goals, using OARS techniques) with these representing similar frequencies.

#### Graduate student qualitative feedback

The graduate students reported various highlights and improvements associated with the Demo, Debrief, and Do (see Table 3). A quote provided by a graduate student on the highlight of the experience includes, “I would see specific students take our feedback and improve on their delivery in the next DDD. I was impressed by the questions they asked and how most of the students were engaged in the process.” Along with this, the graduate students indicated having the opportunity to gain real experience and constructive feedback from the students and professors was a highlight. An example quote of how the experience improved their coaching includes, “The undergraduates provided me the opportunity to reflect on my style of coaching, and to acknowledge the different coaching styles that different people have, and to find ways to accommodate that in different settings/with different clients.” In addition, becoming more secure and confident with the entire coaching process helped to improve their coaching. Although the Demo, Debrief, and Do were designed to provide undergraduate students with the opportunity to gain “real-life” coaching experiences, it is evident that there were beneficial experiences for graduate students as well.

## Discussion

The main objectives of this paper were to describe a SBE approach for health coaching and the need for this teaching method during COVID-19 and describe the impact this technique had on both the undergraduate and graduate students enrolled in a health-related curriculum. This study’s contributions to the literature include highlighting an SBE approach that was successful in its implementation and had a positive impact on the acquired learning in both graduate and undergraduate student populations.

The Demo Debrief and Do (DDD) simulation approach fulfilled an educational need during the COVID 19 pandemic. It also continues to fill a gap in offering simulation-based education opportunities for both graduate and undergraduate students while learning effective client-communication skills for best practice in health coaching delivery in a safe environment. Additionally, students learned how to navigate and communicate within a virtual session, a valuable skill for our future leaders in health care. Overall, we found that this SBE practice had benefits across all participants in the class, the faculty, undergraduate students, and graduate students. This is similar to findings that Jeong and colleagues (2020) found in their implementation of virtual peer teaching related to medical patient education as this program benefited the faculty, students and the peer teachers.

### Impact on undergraduate students

After implementation of the Demo, Debrief, and Do simulation approach, the instructors found that the DDD was highly effective and valuable in teaching benchmarks skills visible in the higher quality coaching showcased in the videos created by the students after the learning experiences. In our study, the undergraduate students indicated, with highest frequency, that the feedback and tips from the graduate students during the debriefing section of the DDD was the highlight of the sessions. They were able to apply this feedback, as well as feedback from their peers to their videos demonstrating their skills. Kourgiantakis and Lee [13] also utilized feedback in their virtual practice Fridays and their students commented that these techniques assisted them in improving their social worker skills. In our study, the undergraduate students indicated that their coaching skills improved and they gained more confidence in their abilities to effectively communicate with individuals related to health behavior change. This is evidenced by the following quote: “working with the health coaches provided me with a demonstration of how the video should run with my partner for the case study. Practicing with my partner in front of the class and coaches allowed me to overcome my fear of acting as a coach. Seeing the other teams perform the same scenario and receive advice also made me feel more comfortable and confident”.

Instructors also noticed improved health coaching skill sets compared to past undergraduate cohorts in similar video assignments. The following quote exemplifies how the DDD was a supportive mechanism in their learning and application of targeted health coaching skills: “My coaching improved by watching their session, discussing what they did right and wrong, and then by practicing it with other people in my group. In the beginning of the zoom call, the health coaches played a video of them in a session with each other, then we all discussed what the health coach did poorly and what they health coach did well, then we applied those observations and practiced with each other”. Overall, demonstrating, debriefing and practicing with the graduate students was valuable as it allowed the undergraduate students an opportunity to “try out” their assignment in a safe environment with their peers and receive real time feedback before attempting to create their own graded video.

Furthermore, the smaller groups may have increased overall engagement with the material. The graduate student to undergraduate student ratio was 2 to 10. Additionally, the undergraduate students reported a greater understanding of the process of health coaching in this format. These in class instructions offered a foundation of coaching skills which was focused on segments of a coaching session and the DDD offered an example of putting the segments together in real time as demonstrated by the following quote: “The highlight of working with the graduate students was getting student interaction with individuals with students in the graduate program and getting their perspective on how they would typically administer a health coaching session. It was nice to learn from other students and get that hands-on experience of what a health coaching session entails and how we can become better with learning the proper tools to be a successful coach.”

The results from The Student Satisfaction and Self-Confidence in Learning survey completed by the students after each DDD experience captured favorable responses indicating that the students learned, practiced and refined the intended skills and strategies with greater confidence, competence and satisfaction. Additionally, the survey results indicate that they were satisfied with the SBE method in which the instructors taught the intended skills. SBE has been shown in literature to promote active learning and improve self-confidence by practicing, in real time, skills and strategies to support client health behavior choices and changes [12–14].

### Impact on graduate students

The graduate coaches also showed improvements in specific areas, such as feeling comfortable with discussing certain topics, the use of effective nonverbal skills, and satisfaction with active listening skills. An example of a graduate student quote is, “one of the skills I worked on improving for myself was my use of affirmations. I find they are the hardest part of OARS to use well, so it was interesting to hear the student's ideas on how to use affirmations and develop some that naturally integrated into the conversation.” The results of this program may indicate that this learning technique can be adapted and used in a variety of educational settings.

### Strengths and limitations

Strengths of this research include using mixed methods approaches to gather both quantitative and qualitative information. An additional strength was the inclusion of multiple perspectives in examining the DDD approach, including undergraduate student, graduate student, and instructor perspectives. Limitations of the DDD include student feedback bias as the student may offer a higher satisfaction rate or positive experience because the assignments and surveys were not anonymous and part of a graded assignment. Also, the information obtained through surveys and reflections capture one cohort of students during one semester of the undergraduate course. Additionally, there are only a few academic programs that offer health coaching training at a graduate level and therefore fewer students possessing the skill set needed to facilitate a DDD experience. To accurately assess the effectiveness of the DDD, additional cohorts of students (graduate and undergraduate) over a course of several semesters would offer more data to measure this SBE teaching method.

## Conclusion

### Lessons learned for practice of innovative higher education

We found that implementation of this SBE experience, the DDD, was easy to implement via a virtual platform, ZOOM. During COVID, the virtual platform was needed and then was continued remotely. This platform allowed the graduate students to participate even though some of them were not close by geographically, while removing the barrier of time constraint. This type of teaching can be continued as part of the course instruction after COVID-19 restriction in both the graduate and undergraduate curriculum.

Even though the SBE experience was conducted virtually, the undergraduate students responded positively to having a graduate student instead of an instructor facilitate the group. These small virtual groups facilitated by the graduate students may have felt more informal and allowed the undergraduate students to feel less intimidated while participating in this safe environment. One student explains: “I was able to practice my skills in a smaller breakout room setting with the coaches, instead of a regular, big class session. This helped me practice my skills while also increasing my confidence levels and learning along the way. Also, being able to view the coaches’ demonstration as well as the peer partner examples, helped to give me better ideas of the possible responses I could encounter and how I would professionally respond to them. For example, how to handle a client that is hesitant to get started or maybe be really nervous to share their concerns with me". The instructors learned through individual undergraduate student reflections and satisfaction of learning surveys that students found value in the DDD experience. Additionally, through standard university course evaluation, several students added in comment sections that the DDD was a highlight of their learning experience. Instructors also gleaned through qualitative feedback from graduate students that first learning the skills through their own coursework, and then demonstrating the skills in front of the undergraduate students, and lastly offering supportive feedback to the undergraduate students during the session aided in their own development and growth.

The DDD has the potential to be implemented not only in teaching health behavioral change skills, but also in other healthcare specialties where SBE is the gold standard. To further support the efficacy of using a DDD style format is evidence of this technique being successfully used with graduate students teaching undergraduate students nutrition concepts; specifically, a dietetics program incorporating health coaching skills [30]. Researchers found an increase in overall knowledge of health coaching in both the graduate student health coaches and the undergraduate participants.

### Future research

Future research may include investigating the implementation of this technique as supplemental modules that can be utilized at other institutions to support best practices in health behavior change curriculum. Furthermore, there is potential for the DDD to be modified and adapted in various academic settings and formats to support best practice for experiential learning of client-centered skill sets needed to work with individuals in a wide variety of healthcare settings and academic fields. With the development of technology and continued advancement of educational initiatives, this type of teaching technique has the potential to be adapted to meet the needs of students pursuing client-centered communication skills in various learning environments and diverse healthcare settings.

### Supplementary Information

**Additional file 1: Supplemental File 1. **Qualitative Survey Questions.

## Data Availability

The datasets used and/or analyzed during the current study are available from the corresponding author on reasonable request.

## References

[1] Moran K, Milsom A (2015). The flipped classroom in counselor education. Couns Educ Superv.

[2] Prince M (2004). Does active learning work? A review of the research. J Eng Educ.

[3] Al-Elq AH (2010). Simulation-based medical teaching and learning. J Fam Community Med.

[4] Lateef F (2010). Simulation-based learning: Just like the real thing. J Emerg Trauma Shock.

[5] Astbury J, Ferguson J, Silverthorne J, Willis S, Schafheutle E (2021). High-fidelity simulation-based education in pre-registration healthcare programmes: a systematic review of reviews to inform collaborative and interprofessional best practice. J Interprof Care.

[6] Ickes MJ, McMullen J (2016). Evaluation of a health coaching experiential learning collaboration with future health promotion professionals. Pedagogy Health Promotion.

[7] Ladyshewsky RK (2010). Building competency in the novice allied health professional through peer coaching. J Allied Health.

[8] Bandura A (1987). Self-efficacy: The exercise of control.

[9] Neuwirth LS, Jović S, Mukherji BR (2021). Reimagining higher education during and post-COVID-19: Challenges and opportunities. J Adult Continuing Educ.

[10] Pace C, Pettit  SK, Barker KS (2020). Best practices in middle level quaranteaching: Strategies, tips and resources amidst COVID-19. Becoming J Georgia Association  Middle Level Educ.

[11] Adnan M, Anwar K (2020). Online Learning amid the COVID-19 Pandemic: Students' Perspectives. Online Submission.

[12] Jeong L, Smith Z, Longino A, Merel SE, McDonough K (2020). Virtual peer teaching during the COVID-19 pandemic. Med Sci Educ.

[13] Kourgiantakis T, Lee E (2020). <? covid19?> Social work practice education and training during the pandemic: Disruptions and discoveries. Int Soc Work.

[14] Malone M, John E, Ridgeway P (2021). Rapid deployment of a virtual nurse residency program; virtually no idea where to start. J Nurses Prof Dev.

[15] Lawson K (2013). The four pillars of health coaching: preserving the heart of a movement. Global Advances Health Med.

[16] Zigmont JJ, Kappus LJ, Sudikoff SN (2011). The 3D Model of Debriefing: Defusing, Discovering, and Deepening. Semin Perinatol.

[17] Nbhwc. “Home.” *NBHWC*, NBHWC, 15 Aug. 2022, https://nbhwc.org/.

[18] Whitney D, Cooperrider D. Appreciative inquiry: A positive revolution in change. Berrett-Koehler Publisher, Inc. 2011.

[19] Miller WR, Rollnick S. Motivational Interviewing: Preparing people for change. Guildford Press; 2002.

[20] Ibrahim M, Callaway R. Students' learning outcomes and self-efficacy perception in a flipped classroom. InE-Learn: World Conference on E-Learning in Corporate, Government, Healthcare, and Higher Education. 2014. pp. 899–908.

[21] Keskitalo T, Ruokamo H. A pedagogical model for simulation-based learning in healthcare. In Seminar. net Journal of Media and Technology and Life Long Learning. 2015;11(2):74–86.

[22] O’Regan S, Molloy E, Watterson L, Nestel D (2016). Observer roles that optimise learning in healthcare simulation education: a systematic review. Adv Simul.

[23] Miller WR, Rollnick S (2013). Motivational Interviewing: Helping people to change (3rd Edition).

[24] Motivational Interviewing Network of Trainers (MINT) website. https://motivationalinterviewing.org/ Accessed July 13, 2023

[25] MINT Website: Understanding Motivational Interviewing https://motivationalinterviewing.org/understanding-motivational-interviewing Accessed July 13, 2023

[26] Rollnick S, Miller W, Butler C (2022). Motivational Interviewing in Health Care: Helping Patients Change Behavior.

[27] National League for Nursing. (2016). Description of available tools. Retrieved from https://www.nln.org/home

[28] Unver V, Basak T, Watts P, Gaioso V, Moss J, Tastan S, Iyigun E, Tosun N (2017). The reliability and validity of three questionnaires: the student satisfaction and self-confidence in learning scale, simulation design scale, and educational practices questionnaire. Contemp Nurse.

[29] Hsieh H-F, Shannon SE (2005). Three Approaches to Qualitative Content Analysis. Qual Health Res.

[30] Sheehan-Smith L, Brinthaupt TM (2010). Using service-learning to teach health coaching. Academic Exchange Quarterly.

