# Supplementary material for: Impact of a simulation-based education approach for health sciences: demo, debrief, and do
Source: BMC Med Educ. 2023 Oct 10;23:747. doi: 10.1186/s12909-023-04655-w (PMC10566061; doi:10.1186/s12909-023-04655-w)
Supplement: Supplementary file 1 — Additional file 1: Supplemental File 1. Qualitative Survey Questions. [file 12909_2023_4655_MOESM1_ESM.docx]

Supplemental File 1

Qualitative Survey Questions

In one page, answer the following questions based on your class session with the Practicum Student Coaches:

1. What was the highlight of working with the student Practicum Health Coaches for you?

2. How did your coaching improve by working with the Practicum Health Coaches?

3. What area of coaching did you feel most comfortable coaching?

4. What felt "uncomfortable" or "hard" when you were coaching?

5. What did you make sure to include in your video based on what you learned in your session with the Practicum Student Coaches?
